# Abdominal Wall in Cross-Sectional Imaging as an Essential Knowledge for Radiologists: A Pictorial Review

**DOI:** 10.7759/cureus.91594

**Published:** 2025-09-04

**Authors:** Andrés Felipe Herrera Ortiz, Valeria Del Castillo, Laura Olarte Bermúdez, Gonzalo A Montaño Rozo, Camilo Soler, Rodrigo Borrero, Oscar Rivero, Diego Aguirre

**Affiliations:** 1 Radiology, Fundación Santa Fe de Bogotá, Bogotá, COL; 2 Radiology, Universidad El Bosque, Bogotá, COL; 3 Faculty of Medicine, Universidad El Bosque, Bogotá, COL; 4 Paediatrics and Child Health, Fundacion Santa Fe de Bogotá, Bogotá, COL; 5 Radiology, Fundación Santa Fé de Bogotá, Bogotá, COL

**Keywords:** abdominal radiology, abdominal wall hernial, abdominal wall metastases, cross-sectional imaging, inflammatory process

## Abstract

The abdominal wall, a complex anatomical structure, is vital in protecting abdominal organs and maintaining intra-abdominal pressure. Given its intricate composition of muscles, fascia, and connective tissues, accurate imaging and interpretation of abdominal wall pathologies are essential for radiologists. Conditions such as hernias, traumatic injuries, infections, and tumors are common yet often challenging to diagnose. This pictorial review aims to provide radiologists with a comprehensive understanding of abdominal wall anatomy and essential findings in cross-sectional imaging across various pathologies. By mastering abdominal wall imaging, radiologists can significantly enhance their diagnostic accuracy and patient care.

## Introduction and background

The abdominal wall, often overlooked in diagnostic radiology, is a complex structure composed of soft tissues that protect and contain abdominal organs. It is delineated superiorly by the costal margins and xiphoid process, inferiorly by the iliac crests, pubis, and inguinal ligaments, and posteriorly by the transverse processes of the lumbar vertebrae [1]. 

Anteriorly, the abdominal wall is composed of the rectus abdominis muscle encased in the rectus sheath. This sheath consists of anterior and posterior layers formed by the aponeuroses of the lateral abdominal muscles. These fasciae converge medially, forming the linea alba along the midline [2]. Laterally, the abdominal wall comprises three key muscles: the external oblique, internal oblique, and transversus abdominis [2].

While ultrasound is the first-line imaging modality for evaluating abdominal wall diseases due to its low cost and wide availability, magnetic resonance imaging (MRI) remains the gold standard. MRI provides superior anatomical detail and tissue characterization, making it the most accurate modality for assessing the abdominal wall [3,4]. 

This pictorial review aims to provide radiologists with a comprehensive understanding of abdominal wall anatomy and essential findings in cross-sectional imaging across various pathologies. 

## Review

Abdominal wall hernias

The majority of abdominal wall hernias are initially evaluated with ultrasound due to its lack of ionizing radiation, low cost, and wide availability. Ultrasound effectively provides information on the hernia sac’s contents, location, and defect size. Cross-sectional imaging is typically reserved for cases where ultrasound is inconclusive, such as obturator hernias, or when complications like bowel obstruction are suspected.

Protocol for Abdominal Wall Hernia Assessment in Cross-Sectional Imaging

Multiplanar computed tomography (CT) imaging is recommended for a thorough evaluation and is typically performed using high-performance, low-dose multidetector scanners equipped with advanced reconstruction algorithms [5]. The use of intravenous or oral contrast is not routine except in situations where there is suspicion of incarceration, strangulation, or the presence of collections [6]. 

**Figure 1:**
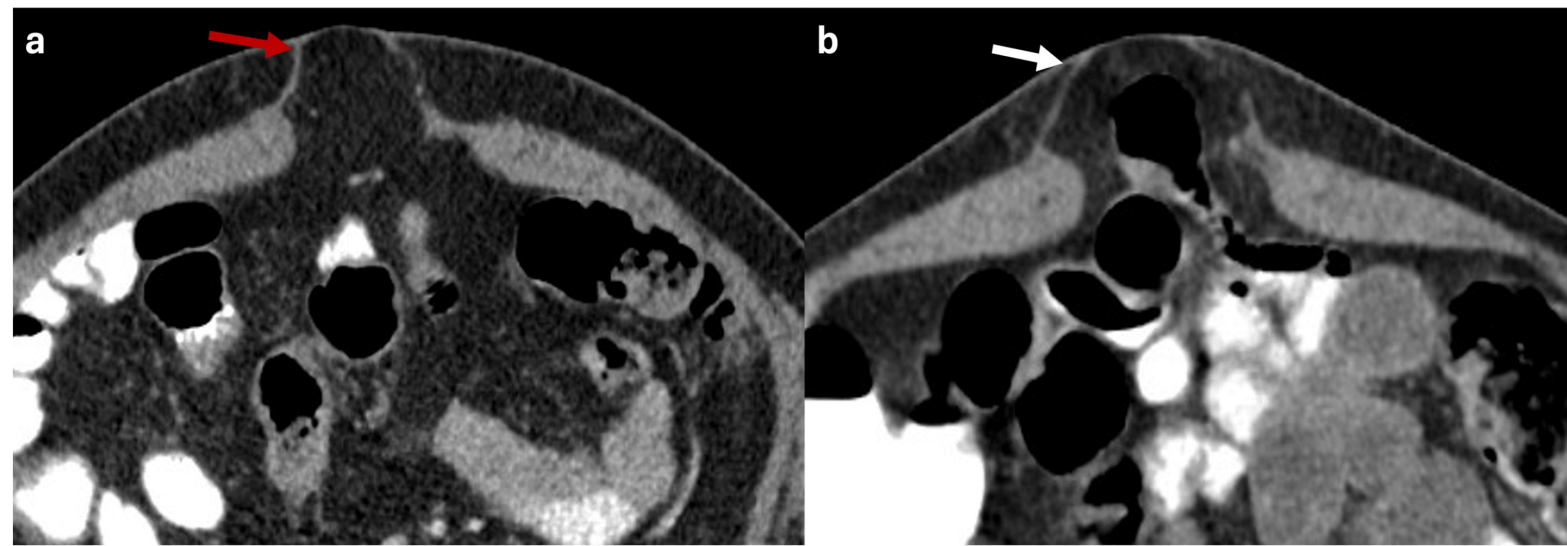
Axial CT scan of the abdomen with oral contrast showing how Valsalva maneuver modifies the content of the hernia sac (a, b) Axial CT scan of the abdomen with oral contrast showing a ventral abdominal wall hernia sac with fat content (red arrow). Image acquisition during the Valsalva maneuver increases the intra-abdominal pressure, showing that the hernia sac is then occupied by small bowel loops (white arrow). The figure was created by the authors.

Likewise, Valsalva‐maneuver imaging is typically reserved for cases with diagnostic uncertainty about the presence of a hernia sac or the nature of its contents. This technique raises intra-abdominal pressure from approximately 6 mmHg to over 20 mmHg, promoting the protrusion of intra-abdominal contents through the abdominal wall defect (Figure 1). However, in most cases, an accurate diagnosis can be made without the need for this maneuver [5]

3D volume-rendered acquisitions offer a more comprehensive and panoramic view of abdominal hernias, often aiding the detection of subtle bulges, atrophic changes, and the position of surgical meshes [5].

Cine-MRI is a valuable tool for evaluating abdominal wall hernias, offering the advantages of being radiation-free and enabling dynamic acquisitions that provide detailed insight into the movement and interaction between the abdominal wall defect and intra-abdominal contents [7].

Joppin et al. proposed an MRI protocol for the evaluation of abdominal hernias in which the patient is positioned supine inside a 3 Tesla MRI scanner, with a flexible body coil placed over the abdomen. The protocol includes a T2 HASTE sequence with 5 mm contiguous slices and a 3D T1 DIXON sequence with 3 mm slices, both acquired in the axial plane during breath-hold after inhalation, covering the region from the pubis to the xiphoid process [7]. Dynamic imaging is acquired using TRUEFISP sequences, with parameters such as field of view and temporal resolution customized to each patient’s body size to optimize image quality and diagnostic accuracy [7].

Indirect Inguinal Hernias

**Figure 2:**
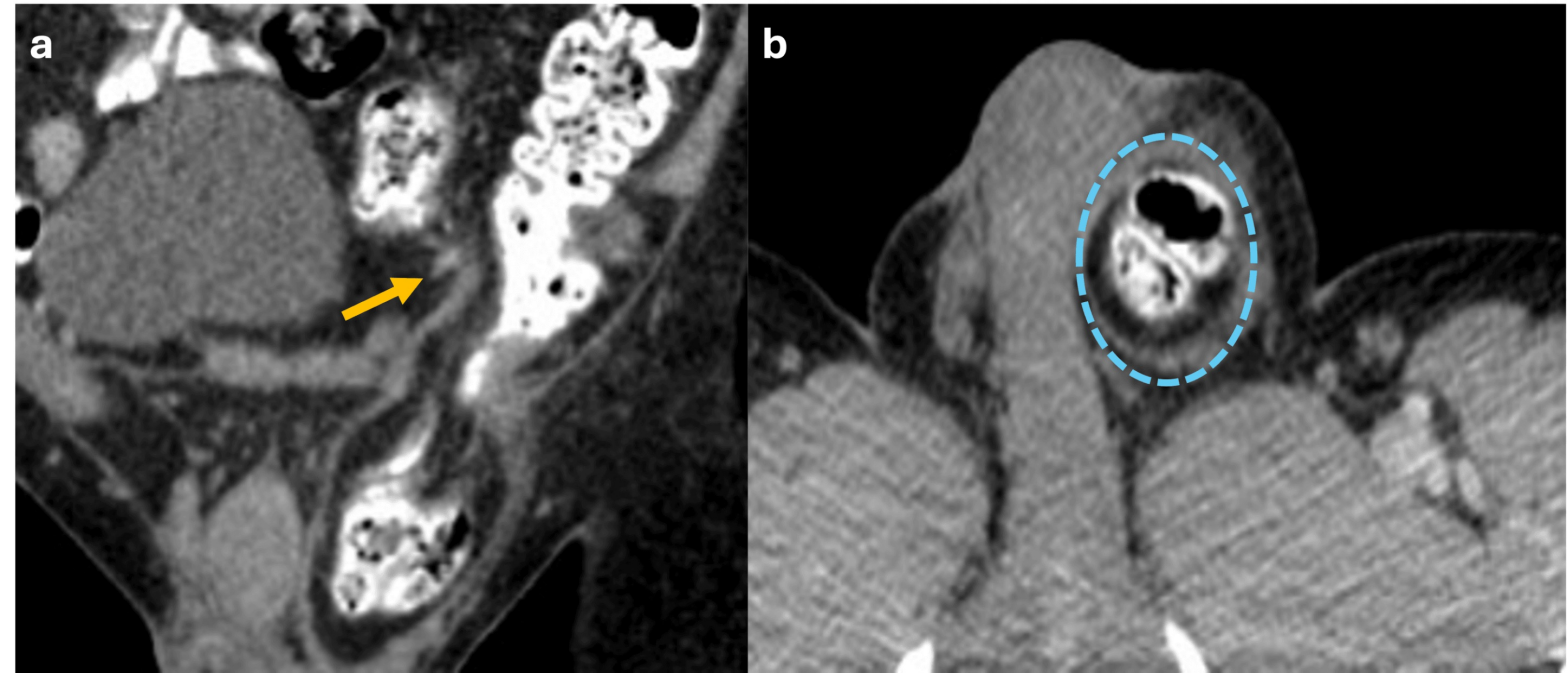
Coronal and axial CT scan of the abdomen with oral contrast showing an indirect inguinal hernia (a) Coronal CT scan of the abdomen with oral contrast revealing an indirect inguinal hernia positioned laterally to the inferior epigastric vessels (yellow arrow). (b) Axial CT scan showing that the hernial sac within the inguinal canal contains part of the sigmoid colon (blue dotted circle). The figure was created by the authors.

Indirect inguinal hernias protrude through the internal inguinal ring and are the most common in adults and children [6]. In adults, it typically originates from the dilation of the internal inguinal ring due to weakness in the wall, being more prevalent in men than in women [6]​​​​​​​. Conversely, in children, this hernia is generally caused by a patent processus vaginalis. On CT, the hernia is lateral to the inferior epigastric vessels and can occasionally be identified within the inguinal canal, with a notable moderate risk of incarceration (Figure 2). This type of hernia may require urgent surgical evaluation, especially if there is a significant risk of herniated organs becoming trapped and potentially leading to strangulation [​​​​​​​6].

Direct Inguinal Hernias

**Figure 3:**
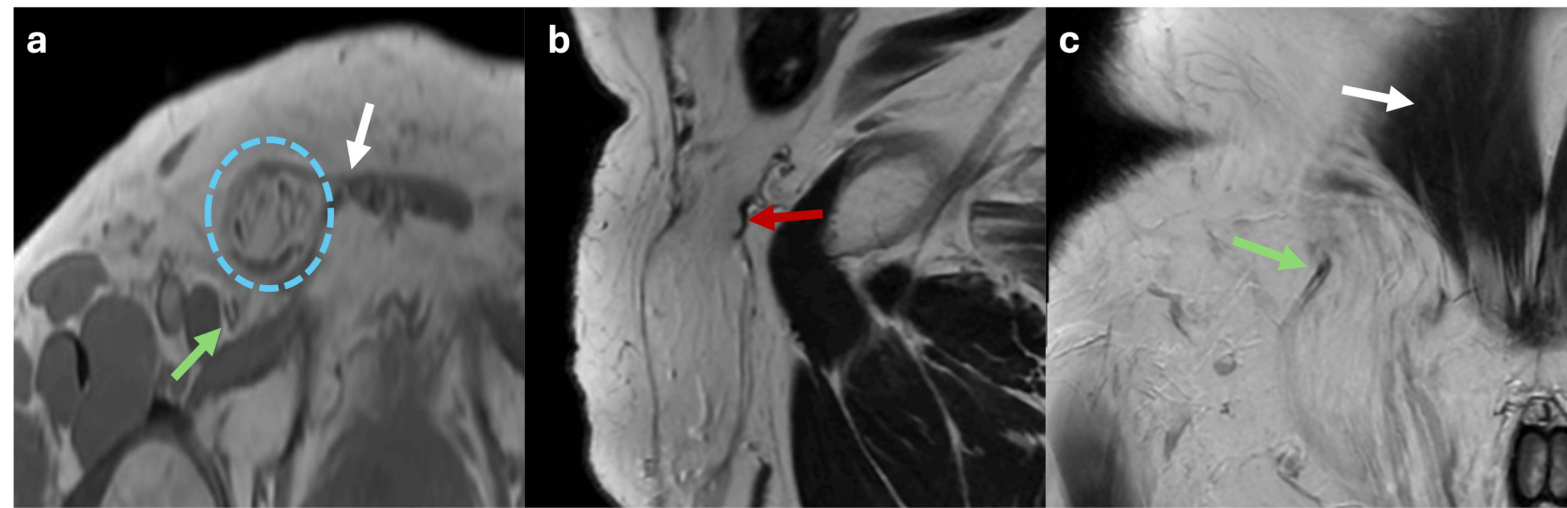
Axial, sagittal and coronal abdominal MRI in T1 and T2 sequences showing a direct inguinal hernia Axial T1 (a), sagittal and coronal T2 (b, c) MRI showing a hernial sac containing fat (blue dotted circle) positioned medial to the inferior epigastric vessels (green arrows), superior to the inguinal ligament (red arrow), and lateral to the rectus abdominis muscle (white arrows) within the Hesselbach’s triangle, consistent with a direct inguinal hernia. The figure was created by the authors.

Direct inguinal hernias in adults typically result from weakness of the transversalis fascia; this condition is more prevalent in men than women [6]. Direct inguinal hernias are located within Hesselbach’s triangle, with its boundaries defined by the inferior epigastric vessels laterally, the inguinal ligament inferiorly, and the rectus abdominis muscle medially (Figure 3). The risk of incarceration of the abdominal contents is generally low [6].

Pantaloon Inguinal Hernias

**Figure 4:**
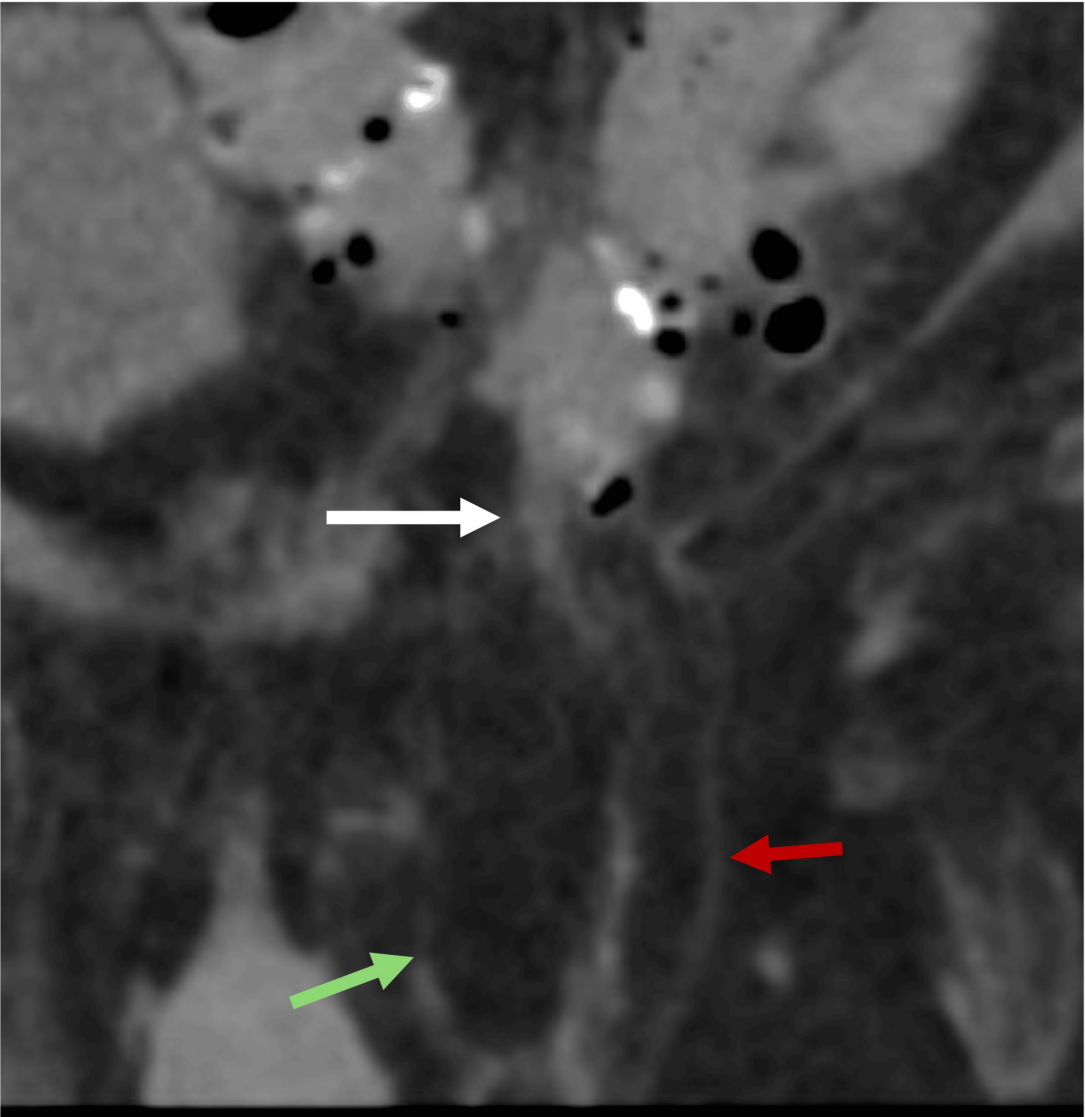
Coronal abdominal CT showing a pantaloon inguinal hernia Coronal abdominal CT showing a PIH with fat content. The direct hernia sac (green arrow) and indirect hernia sac (red arrow) are separated by the  inferior epigastric vessels (white arrow). The figure was created by the authors.

Pantaloon inguinal hernias (PIHs), also called saddlebag hernias, refer to the combination of direct and indirect inguinal hernias on the same side (Figure 4) [8]. PIHs have an incidence ranging from 0.12% to 12.8% among all inguinal hernias [8]. Radiologists must report PIH since it confers a higher likelihood of recurrence after surgery; therefore, the treatment may involve laparoscopic iliopubic tract repair + transabdominal preperitoneal hernioplasty [8]. 

Femoral Hernias

**Figure 5:**
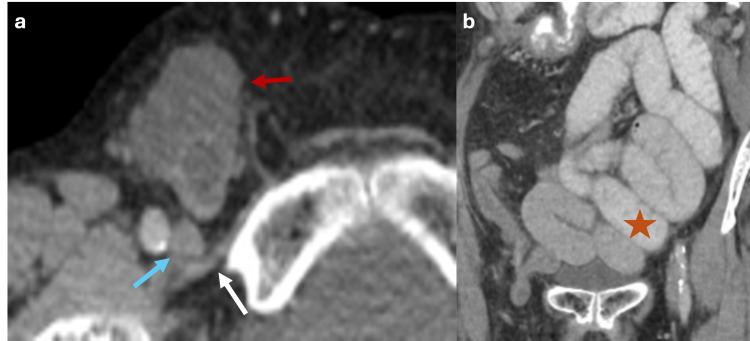
Axial and coronal CT scan of the abdomen with oral contrast showing a femoral hernia with small bowel obstruction (a, b) Axial and coronal CT scan of the abdomen with oral contrast displaying a femoral hernia with fluid and small bowel content (red arrow) located anterior to the pectineus muscle (white arrow) and medial to the femoral vein (blue arrow), leading to bowel obstruction (red star). The figure was created by the authors.

Femoral hernias are located anterior to the pectineus muscle, below the inguinal ligament, and medial to the common femoral vein (Figure 5) [9]. They often compress the femoral vein, a finding known as the femoral vein comma sign [10]. They are four times more common in women than men, probably secondary to dilatation of the femoral ring connective tissue due to hormonal changes or pregnancy [10]. Femoral hernias carry a high risk of incarceration [6].

Ventral Hernias

**Figure 6:**
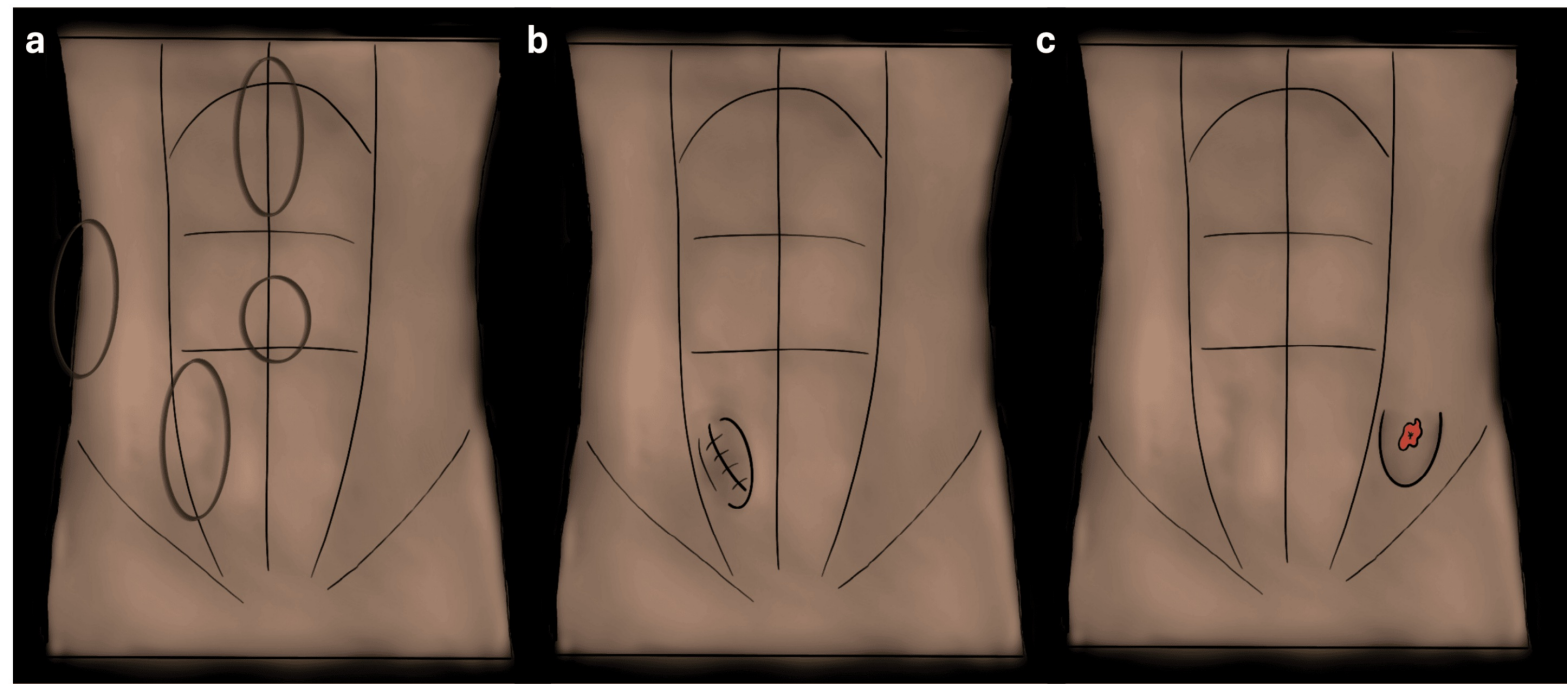
Illustration showing types of ventral hernias (primary ventral hernia, Incisional hernia and parastomal hernia) (a) Primary ventral hernia subtypes can be divided into epigastric, umbilical, Spiegel, and lumbar hernias. (b) Incisional hernia, and (c) parastomal hernia. Illustrations elaborated by the authors.

Ventral hernias are broadly classified into three types: 1) primary hernias, 2) incisional hernias, and 3) parastomal hernias (Figure 6) [11]. Primary hernias, which include epigastric hernias, umbilical hernias, Spiegel hernias, and lumbar hernias (as named by Grynfelt and Petit), can be further categorized based on their size as small (<2 cm), medium (2-4 cm), and large (>4 cm) [11]. Incisional hernias occur in a surgical incision scar, and parastomal hernias are protrusions of abdominal content through a defect adjacent to a stoma [6].

Epigastric Hernias

**Figure 7:**
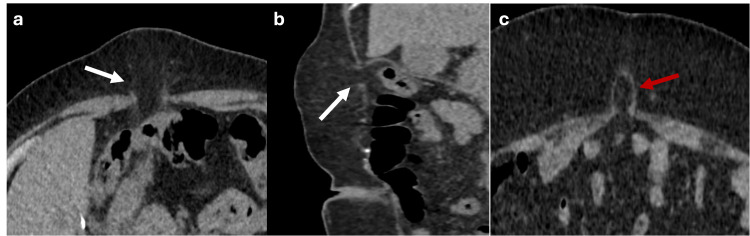
Axial and sagittal non-contrast abdominal CT scan showing an epigastric and umbilical hernia (a, b) Axial and sagittal CT showing a fat-content epigastric hernia located between the xiphoid process and the umbilicus (white arrows). (c) Axial non-contrast abdominal CT scan showing a hernia sac with fat content protruding from the umbilical ring (red arrow), consistent with an umbilical hernia. The figure was created by the authors.

Epigastric hernias, also known as fatty hernias of the linea alba, occur between the xiphoid process and the umbilicus (Figure 7a, 7b). These hernias often occur in pregnant and obese patients [6,12]. They typically contain preperitoneal fat, blood vessels, and rarely abdominal organs such as bowel or stomach [12]. These hernias carry a high risk of incarceration [12].

Umbilical Hernias

Umbilical hernias are the most common ventral abdominal hernias; they occur when the abdominal contents protrude through the umbilical ring (Figure 7c). They are more prevalent in women than men, with a ratio of 10 to 1 [12]. In adults, umbilical hernias are typically congenital and result from incomplete abdominal wall closure after the umbilical cord is tied off. On the other hand, acquired umbilical hernias commonly occur in obese, cirrhotic, and pregnant patients due to increased intra-abdominal pressure. These hernias pose a high risk of incarceration [12]. 

Spigelian Hernias

**Figure 8:**
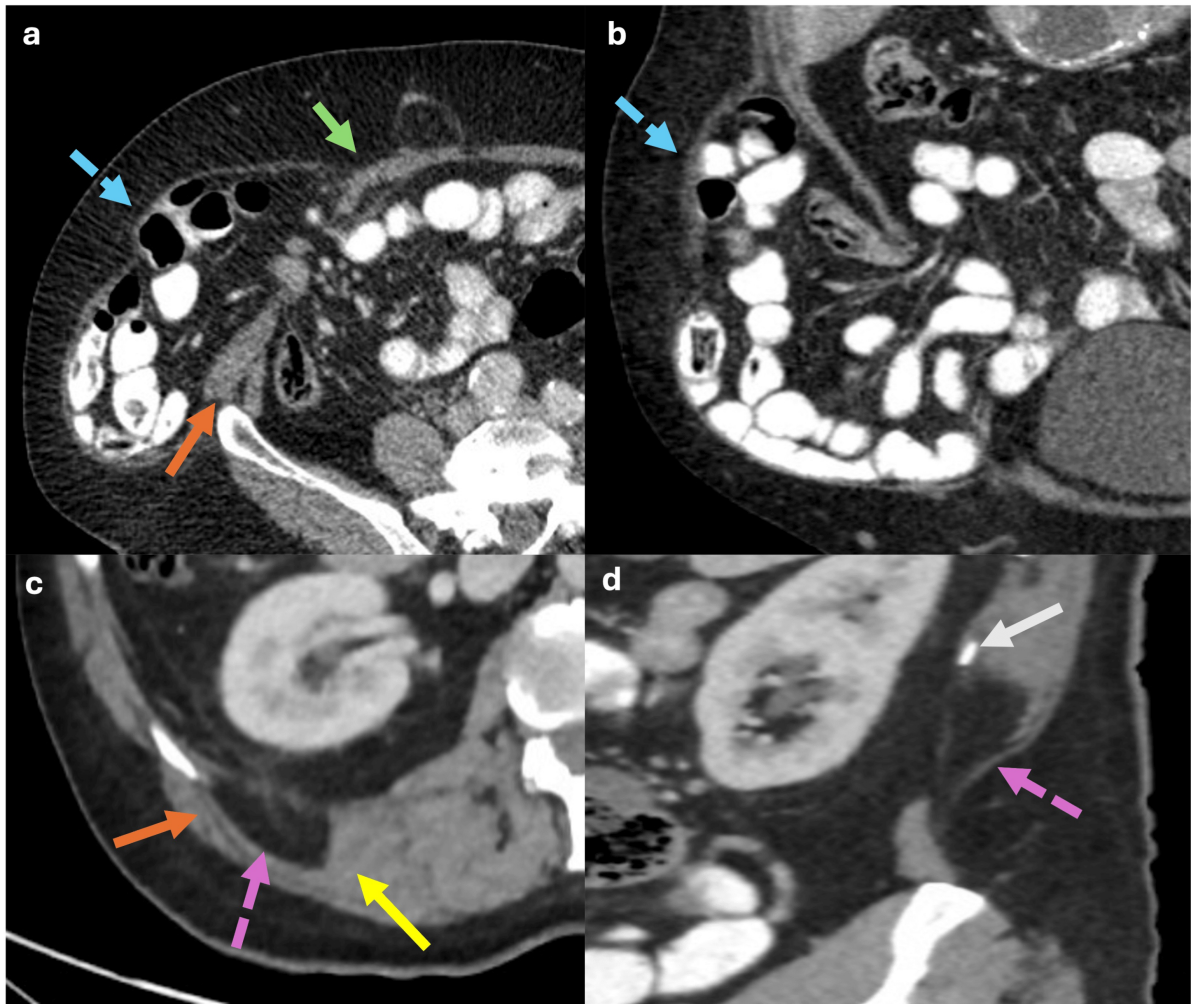
Axial and coronal abdominal CT scan with oral contrast showing a Spigelian hernia and Grynfelt hernia (a, b) Axial and coronal CT scan showing a hernia sac with small bowel loop content (blue dotted arrows) protruding between the rectus abdominis muscle (green arrow) and the internal oblique muscle (orange arrow), consistent with a Spigelian hernia. (c, d) Axial and coronal CT scan from another patient showing a hernia sac with fat content (purple dotted arrows) delimited laterally by the internal oblique muscle (orange arrow), medially by the quadratus lumborum muscle (yellow arrow), and the superiorly by the 12th rib (white arrow) consistent with a Grynfelt hernia in the upper lumbar triangle. The figure was created by the authors.

Spigelian hernias typically occur in patients between 40 and 70 years [13]. They are located between the rectus abdominis muscle and the internal oblique muscle at a point known as the semilunar line. These hernias are usually found in the lower abdomen, where the fascia is weaker (Figures 8a, 8b) [13]. The external oblique muscle often prevents Spigelian hernias from causing a noticeable bulge in the abdominal wall; however, these hernias pose a high risk of incarceration [13]. Up to 75% of Spigelian hernias in male infants are associated with ipsilateral cryptorchidism, probably due to a failure in the development of the gubernaculum [14].

Grynfelt Hernias

Grynfelt hernias commonly occur spontaneously in males between 50 and 70 years. These hernias are located in the superior lumbar triangle, bounded by the internal oblique muscle laterally, the quadratus lumborum muscle medially, and the 12th rib superiorly (Figure 8c, 8d). There is a high risk of incarceration where abdominal contents become trapped within the hernia sac [6].

Petit Hernias

**Figure 9:**
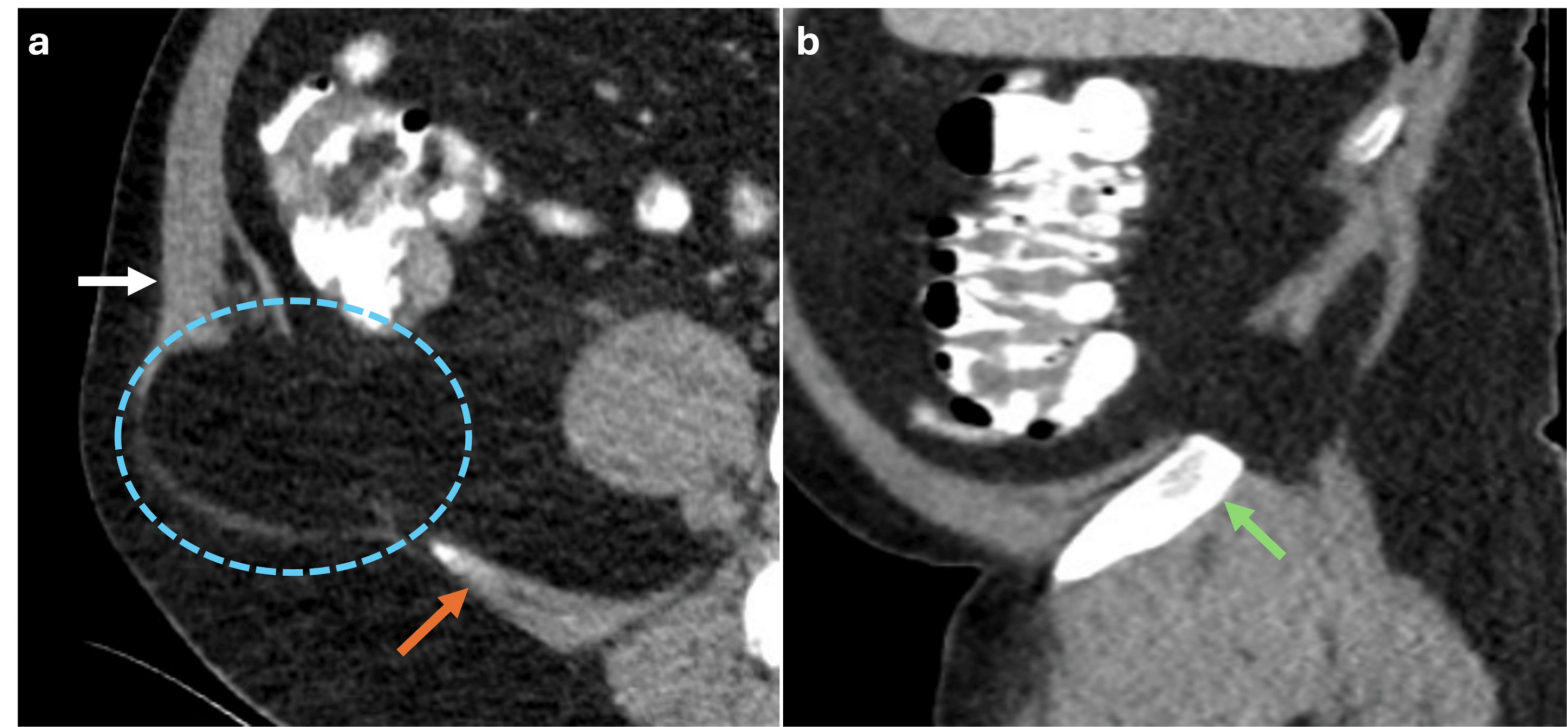
Axial and sagittal abdominal CT scan with oral contrast showing a Petit hernia (a, b) Axial and sagittal CT scan demonstrates a hernia sac with fat content (blue dotted circle) delimited laterally by the external oblique muscle (white arrow), medially by the latissimus dorsi muscle (orange arrow) and inferiorly by the iliac crest (green arrow), consistent with a Petit hernia in the lower lumbar triangle. The figure was created by the authors.

Petit hernias commonly occur spontaneously in males between 50 and 70 years. These hernias are situated in the inferior lumbar triangle, with boundaries defined by the external oblique muscle laterally, the latissimus dorsi muscle medially, and the iliac crest inferiorly (Figure 9). Petit hernias have a high risk of incarceration, where abdominal contents become trapped within the hernia sac [6].

Incisional Hernias

**Figure 10:**
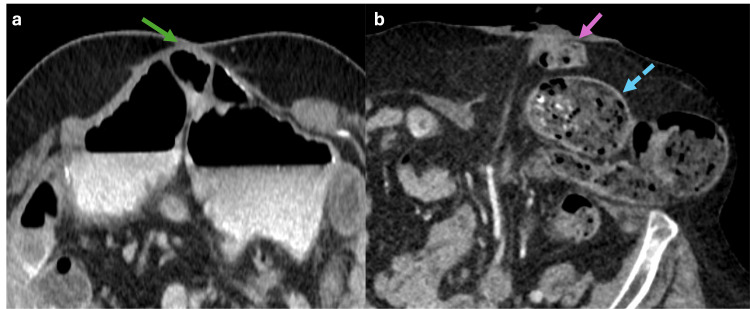
Axial abdominal CT scan with oral contrast showing an incisional hernia and parastomal hernia (a) Axial CT scan showing an anterior abdominal wall weakness with protrusion of transverse colon (green arrow) in a patient that underwent laparotomy; these findings consist of an incisional hernia. (b) Axial abdominal CT scan from another patient showing a colostomy stoma (pink arrow) with a parastomal protrusion of the descending colon (blue dotted arrow) consistent with a parastomal hernia with no signs of bowel obstruction. The figure was created by the authors.

Incisional hernias are a complication that occurs in a previous laparotomy scar within the first few months following abdominal surgery. They are more commonly associated with vertical incisions rather than horizontal ones. These hernias can manifest in almost any location; according to the European Hernia Society (EHS), incisional hernias can be classified into subxiphoid, epigastric, umbilical, infraumbilical, suprapubic, subcostal, flank, iliac fossa, and lumbar regions (Figure 10a) [6,11].

Parastomal Hernias

It is the protrusion of abdominal contents through a defect adjacent to the stoma (Figure 10b) [15]. Essential factors to report include associated intestinal obstruction and growth of the hernia sac compared to the last imaging assessment. The classification consists of the following: type Ia (the bowel forming the colostomy has a sac <5 cm (pre-hernia state)), type Ib (the bowel forming the colostomy has a sac >5 cm), type II (the parastomal hernia sac contains omentum), and type III (the parastomal hernia sac contains bowel loops different from the one forming the stoma) [15].

Obturator Hernias

**Figure 11:**
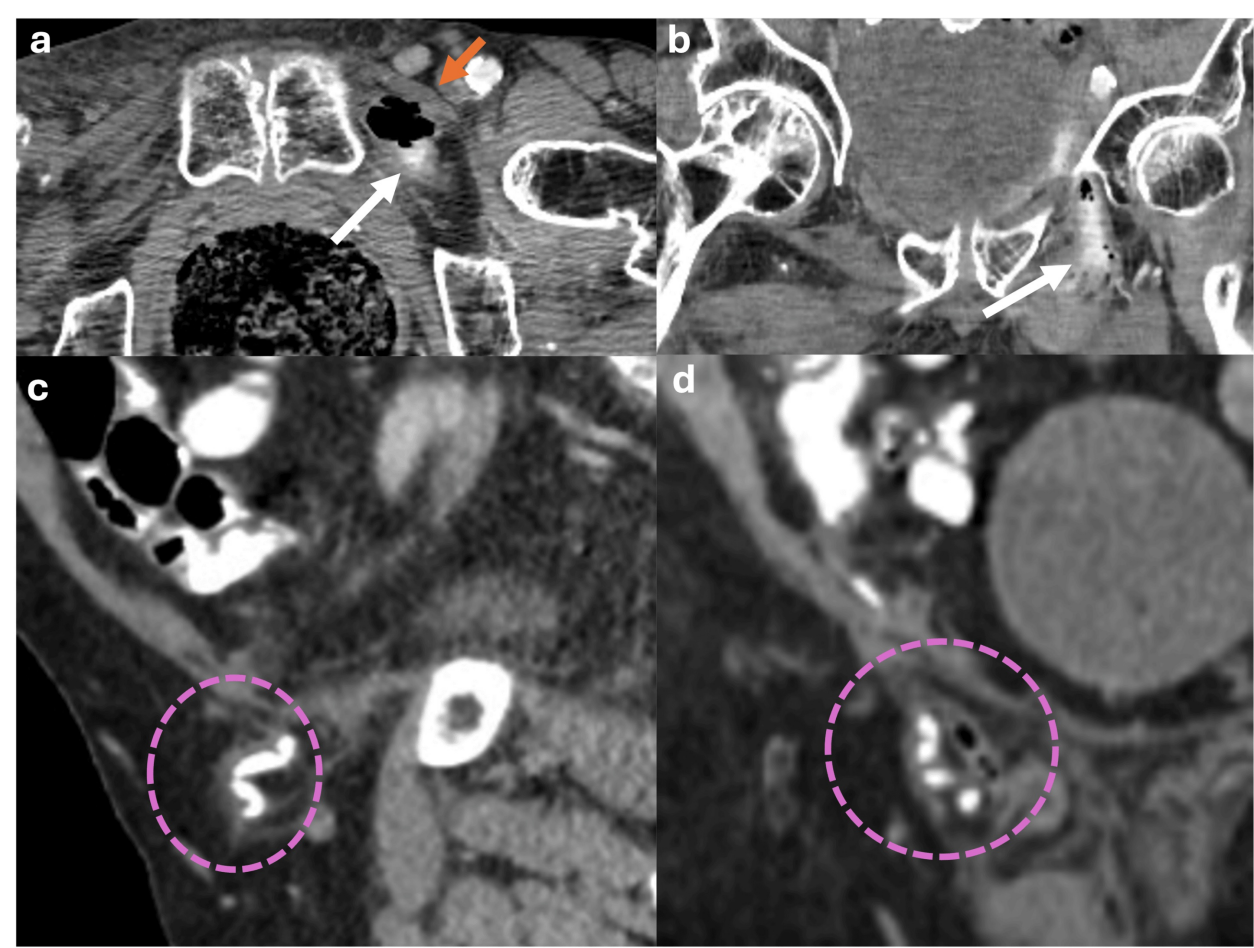
Axial, coronal and sagital abdominal CT scan with oral contrast showing an obturator hernia and Amyand's hernia. (a,b) Axial and coronal CT scan reveals a hernia sac protruding through the obturator foramen (white arrows) located posterior to the pectineus muscle (orange arrow) consistent with an obturator hernia. (c, d) Sagittal and coronal abdominal CT scan from another patient showing a non-inflamed cecal appendix filled with contrast medium, protruding through the inguinal canal consistent with an Amyand's hernia (pink dotted circle). The figure was created by the authors.

Obturator hernias occur when the contents of the abdomen protrude through the obturator foramen posterior to the pectineus muscle (Figure 11a, 11b), potentially leading to compression of the obturator nerve, causing obturator nerve neuropathy. These hernias are more common in elderly females and can be very dangerous, as they are at a high risk of becoming incarcerated [16]. 

Amyand Hernias

Amyand hernias are inguinal hernias that contain the cecal appendix and account for only 1% of all inguinal hernias (Figure 11c, 11d). The appendix can become inflamed within the inguinal canal, occurring in 0.1% of all Amyand hernias [17].

Other Types of Hernias

**Figure 12:**
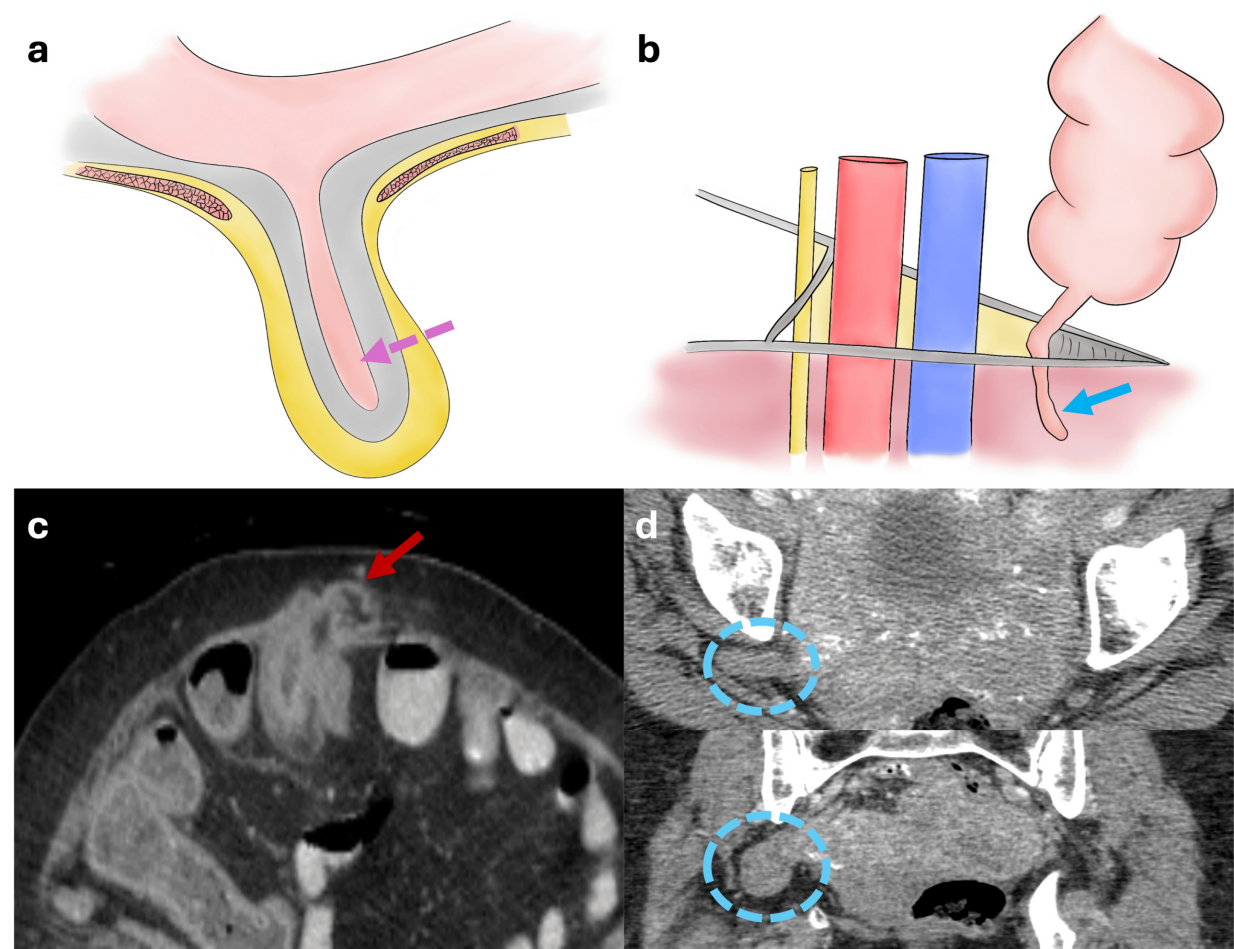
Illustrations depicting other types of hernias (Littre's hernia, Garengeot's hernia, Richter's hernia and Sciatic hernia) (a) Littre's hernia: A Meckel's diverticulum protrudes through an abdominal wall defect (pink dotted arrow). (b) Garengeot's hernia: The cecal appendix protrudes through the femoral canal (blue arrow). (c) Richter's hernia: The antimesenteric border of a short segment of small bowel protrudes through an abdominal wall defect associated with perienteric fat stranding due to strangulation (red arrow). (d) Sciatic hernia: A hernial sac protruding through the sciatic foramen (blue dotted circles). Illustrations elaborated by the authors.

A Littre hernia refers to any hernia containing a Meckel's diverticulum (Figure 12a) [6]. Garengeot’s hernia, on the other hand, is a femoral hernia containing the cecal appendix (Figure 12b) [6]. A Richter hernia involves the herniation of only the antimesenteric edge of the intestine without compromising the entire circumference of the loop, posing a high risk of incarceration (Figure 12c) [6]. Lastly, a sciatic hernia occurs through the sciatic foramen, often due to piriformis muscle atrophy, with typical contents including bowel loops and the ureter (Figure 12d) [6].

Diastasis of the Rectus Abdominis Muscle

**Figure 13:**
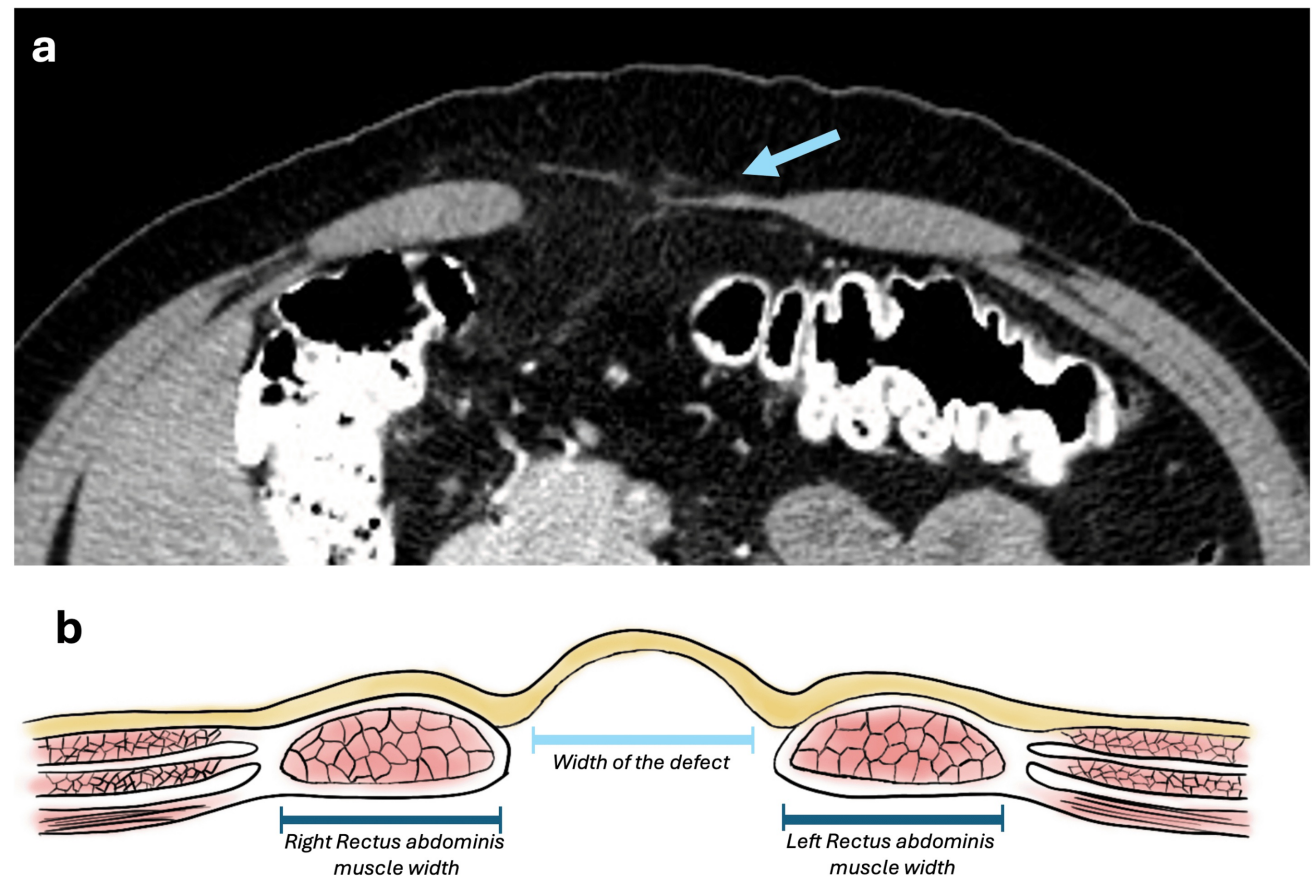
Axial CT scan showing abdominal muscle diastasis and rectus-to-defect ratio (a) Axial CT scan demonstrates thinning of the linea alba with a separation of the medial edges of the rectus abdominis muscles >20 mm consistent with abdominal muscle diastasis (blue arrow). (b) Figure depicting the rectus-to-defect ratio. Illustrations elaborated by the authors.

Diastasis of the rectus abdominis muscles is characterized by thinning of the linea alba with a distance greater than 20 mm between the medial edges of the rectus abdominis muscles [18]. Its significance lies in its association with an increased risk of recurrence in midline ventral hernia repair. The rectus-to-defect ratio (RDR) is a metric used as a predictor of surgical success in repairing midline hernias [18]. It is calculated by dividing the sum of the right and left rectus widths by the defect width (Figure 13). An RDR greater than 2 indicates a success rate of over 90% with standard surgical repair, while an RDR below 1.5 requires component separation techniques in over 52% of cases [18].

Tanaka Method

**Figure 14:**
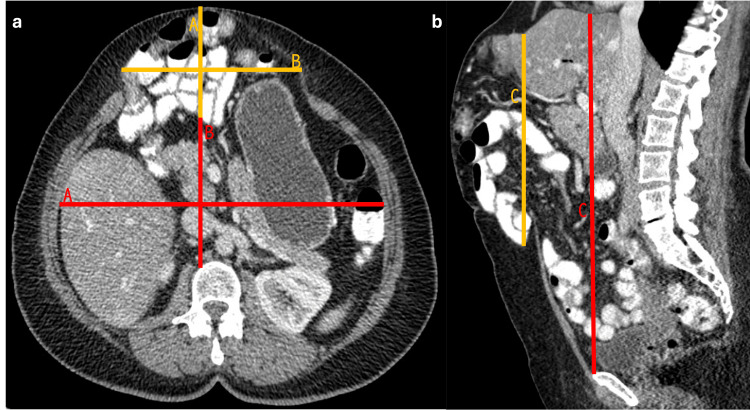
Axial and sagittal abdominal CT scan showing the Tanaka Method (a, b) Axial and sagittal CT scan showing the anteroposterior (A lines), transverse (B lines), and longitudinal (C lines) dimensions of the hernia sac and abdominal cavity to calculate volumes and then apply the Tanaka method. The figure was created by the authors.

The Tanaka method evaluates the volume of the hernia sac and the volume of the abdominal cavity to predict the risk of compartment syndrome when correcting the hernia [19]. The Tanaka method involves dividing the maximum volume of the sac by the maximum volume of the abdominal cavity (Figure 14). If the volume of the hernia sac exceeds 25% of the volume of the abdominal cavity, there is a high risk of compartment syndrome upon hernia correction [19].

Inflammatory processes of the abdominal wall

Abdominal Wall Abscesses

**Figure 15:**
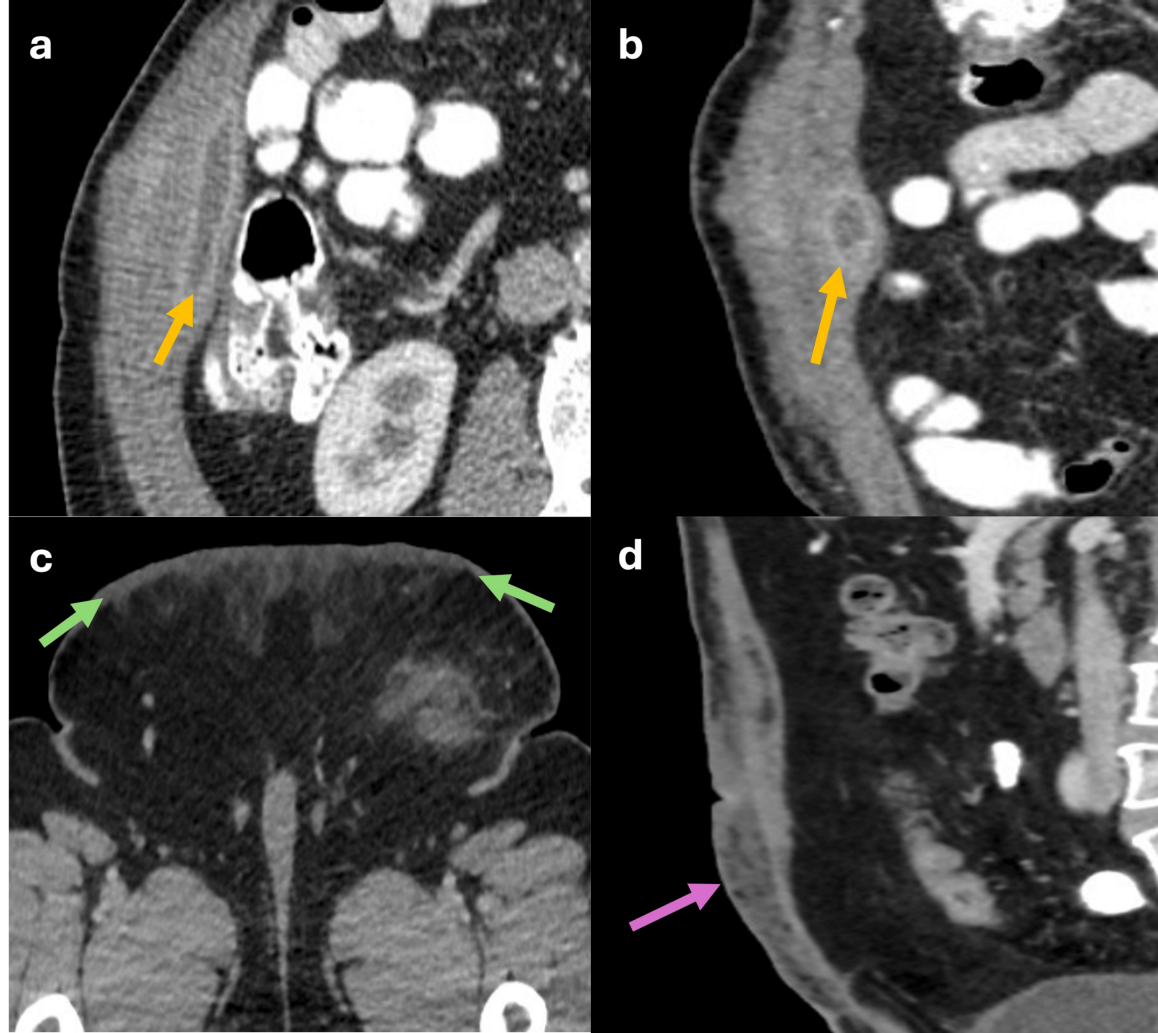
Axial, coronal, and sagittal abdominal CT scan showing an intramuscular abscess and cellulitis (a, b) Axial and coronal CT shows an intramuscular rim enhancing fluid collection inside the right transversus muscle consistent with an abscess (yellow arrows). (c, d) Axial and sagittal CT scan showing thickening of the skin and subcutaneous tissue (green arrows) along with fat stranding (pink arrow), consistent with cellulitis. The figure was created by the authors.

Abscesses of the abdominal wall are characterized by liquid collections with enhancing walls, adjacent fat stranding, and occasionally air bubbles (Figure 15a, 15b). These abscesses can arise post-surgically or secondary to intra-abdominal inflammatory processes that extend to the wall [20].

Abdominal Wall Cellulitis

It presents as skin thickening and increased subcutaneous fat stranding (Figure 15c, 15d). The treatment primarily involves medical management with antibiotics [20].

Enteroatmospheric Fistulas

Entero-atmospheric fistulas are abnormal communications between the gastrointestinal tract and the skin [21]. They can result from various causes, with abdominal surgery being the leading factor, accounting for 80% of cases. Other causes include chronic inflammatory diseases such as Crohn's disease, radiation enteritis, and gastrointestinal tumors; the last is typically associated with superinfection [21]. 

**Figure 16:**
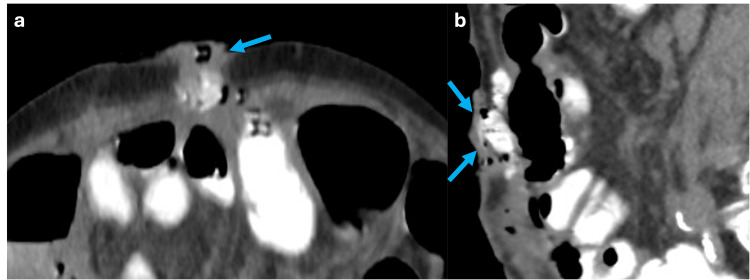
Axial and sagittal abdominal CT scan with oral contrast showing an enteroatmospheric fistula (a, b) Axial and sagittal CT scan shows a fistulous tract communicating the small bowel with the abdominal wall in the umbilical region, consistent with an enteroatmospheric fistula (blue arrows). The figure was created by the authors.

On CT with oral contrast, an entero-atmospheric fistula is seen as a tract filled with contrast that communicates with the skin (Figure 16) [21]. On MRI, an acute fistulous tract typically appears hyperintense on T2-weighted images due to fluid accumulation, and the surrounding mucosa may show a tram-track enhancement after gadolinium administration [21]. As the fistula becomes chronic, it undergoes fibrotic changes, resulting in reduced enhancement and a less prominent T2 hyperintensity due to decreased fluid content [21].

Abdominal Wall Hematomas

**Figure 17:**
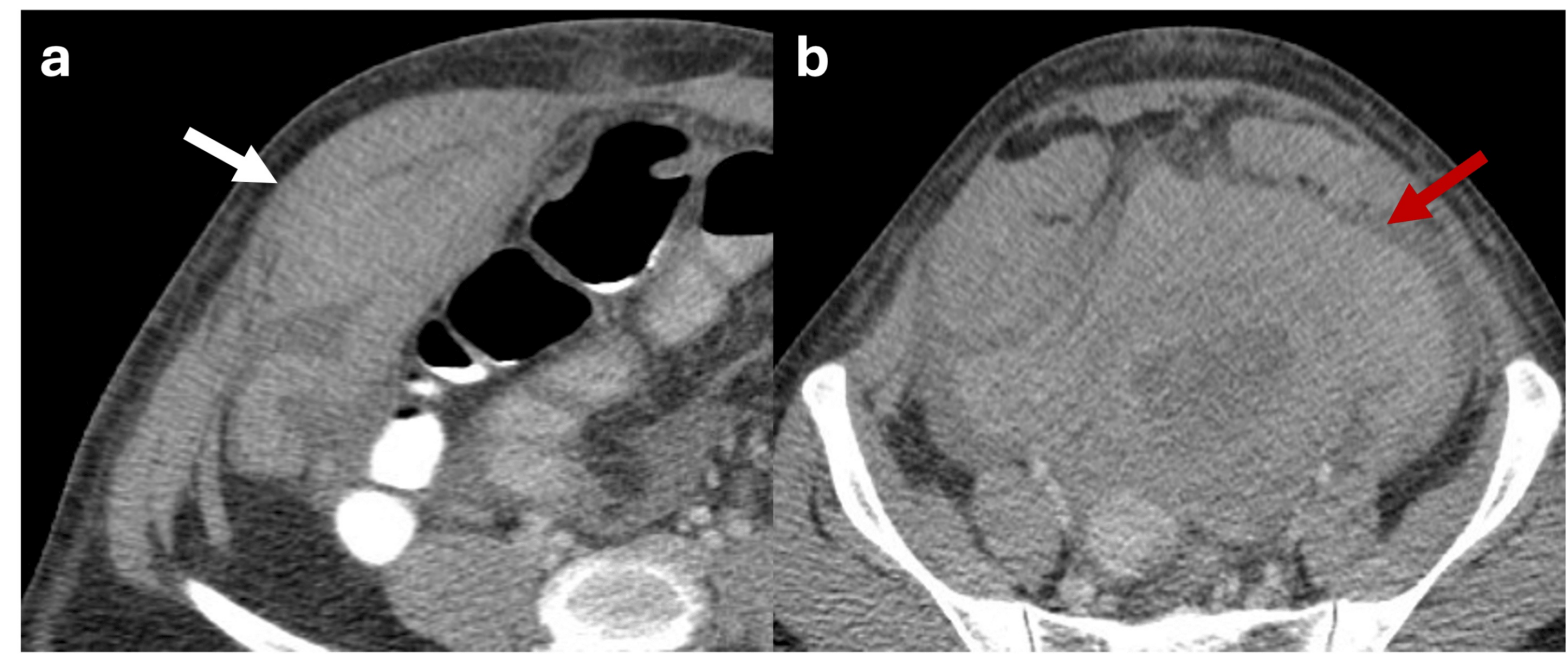
Axial abdominal CT scan showing a rectus abdominis muscle hematoma causing hemoperitoneum (a, b) Axial CT scan shows a hyperdense, non-enhancing lenticular collection within the right rectus abdominis muscle, corresponding to a hematoma (white arrow), notice that the hematoma extends below the arcuate line, rupturing into the abdominal cavity, causing hemoperitoneum (red arrow). The figure was created by the authors.

Abdominal wall hematomas are typically caused by muscle fiber tears or vascular injuries in patients under anticoagulation. On CT scans, they appear as a hyperdense lenticular intramuscular collection without contrast enhancement, resolving in subsequent studies (Figure 17a). When hematomas are located above the arcuate line, they tend to remain confined to the muscle fascia, while those below the arcuate line can extend into the peritoneal cavity (Figure 17b) [20]. 

Endometriosis of the Abdominal Wall

**Figure 18:**
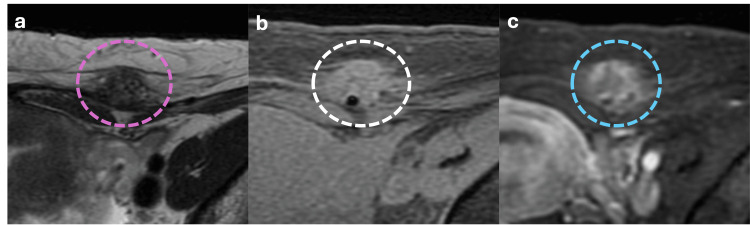
Axial abdominal MRI showing abdominal wall endometriosis deposits (a, b, c) Axial MRI showing a T2 hypointense and T1 slightly hyperintense lesion in the anterior abdominal wall (pink and white dotted circles, respectively), with delayed contrast enhancement after contrast medium administration (blue dotted circle); these findings in the context of a young female with history of cesarean section are highly suspicious for endometriosis deposits. The figure was created by the authors.

Abdominal wall endometriosis typically occurs along scar tissue or laparoscopy access ports following surgeries involving uterine incisions, such as cesarean sections [22]. Pain at the site of endometriosis coincides with menstruation in 50% of cases. On MRI, the lesions appear hypointense on T2-weighted images, hypointense on T1-weighted images (sometimes with internal hyperintense T1 foci of blood), and show delayed contrast enhancement on post-gadolinium images (Figure 18) [22].

Abdominal Wall Varices

**Figure 19:**
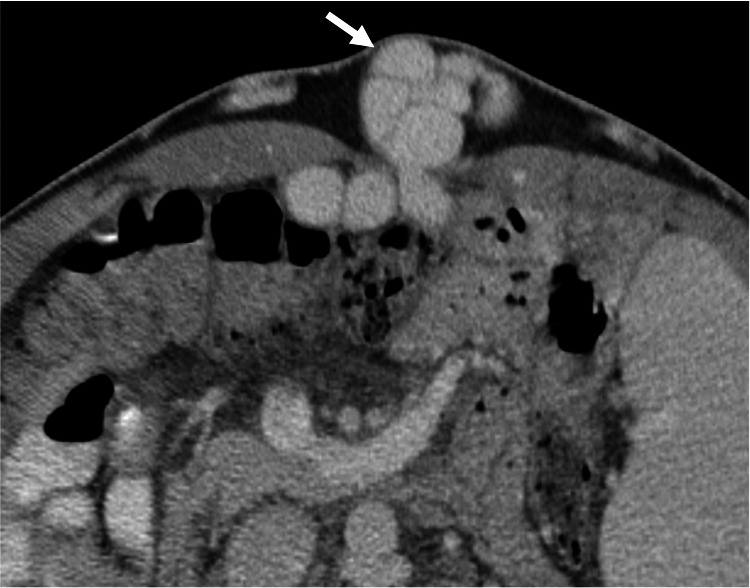
Axial contrast-enhanced abdominal CT scan in the venous phase showing abdominal wall varices Axial CT scan shows multiple dilated and tortuous venous structures with a diameter larger than 3 mm located in the anterior abdominal wall (white arrow); these findings are compatible with abdominal wall varices.

Abdominal wall varices are those measuring >2-3 mm in diameter (Figure 19) [23]. They typically develop in patients with severe portal hypertension, leading to recanalization of paraumbilical veins and formation of subcutaneous varices. These abdominal wall varices frequently anastomose with the superior epigastric and internal mammary veins [23].

Abdominal wall tumors

Abdominal Wall Lipoma

**Figure 20:**
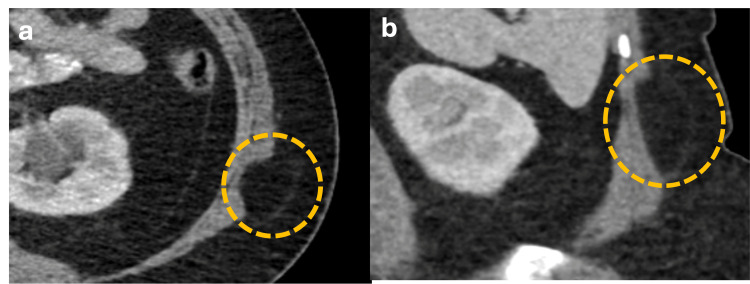
Axial and coronal abdominal CT scan showing an abdominal wall lipoma (a, b) Axial and coronal CT scan showing a hypodense well defined fatty mass within the abdominal wall (yellow dotted circle), consistent with a lipoma. The figure was created by the authors.

Lipoma is the most common benign tumor of the abdominal wall. On CT scans, it appears as an encapsulated tumor with fatty density (Hounsfield units: -10 to -100) (Figure 20). It can be located either subcutaneously or intramuscularly. Occasionally, lipomas may exhibit thin septa (<2 mm) or calcifications [22]. Lipoma-like lesions larger than 10 cm, thick septa (>2 mm), invasion to deep structures, and interval growth may require biopsy due to the likelihood of being a well-differentiated liposarcoma [24].

Desmoid Tumor of the Abdominal Wall

**Figure 21:**
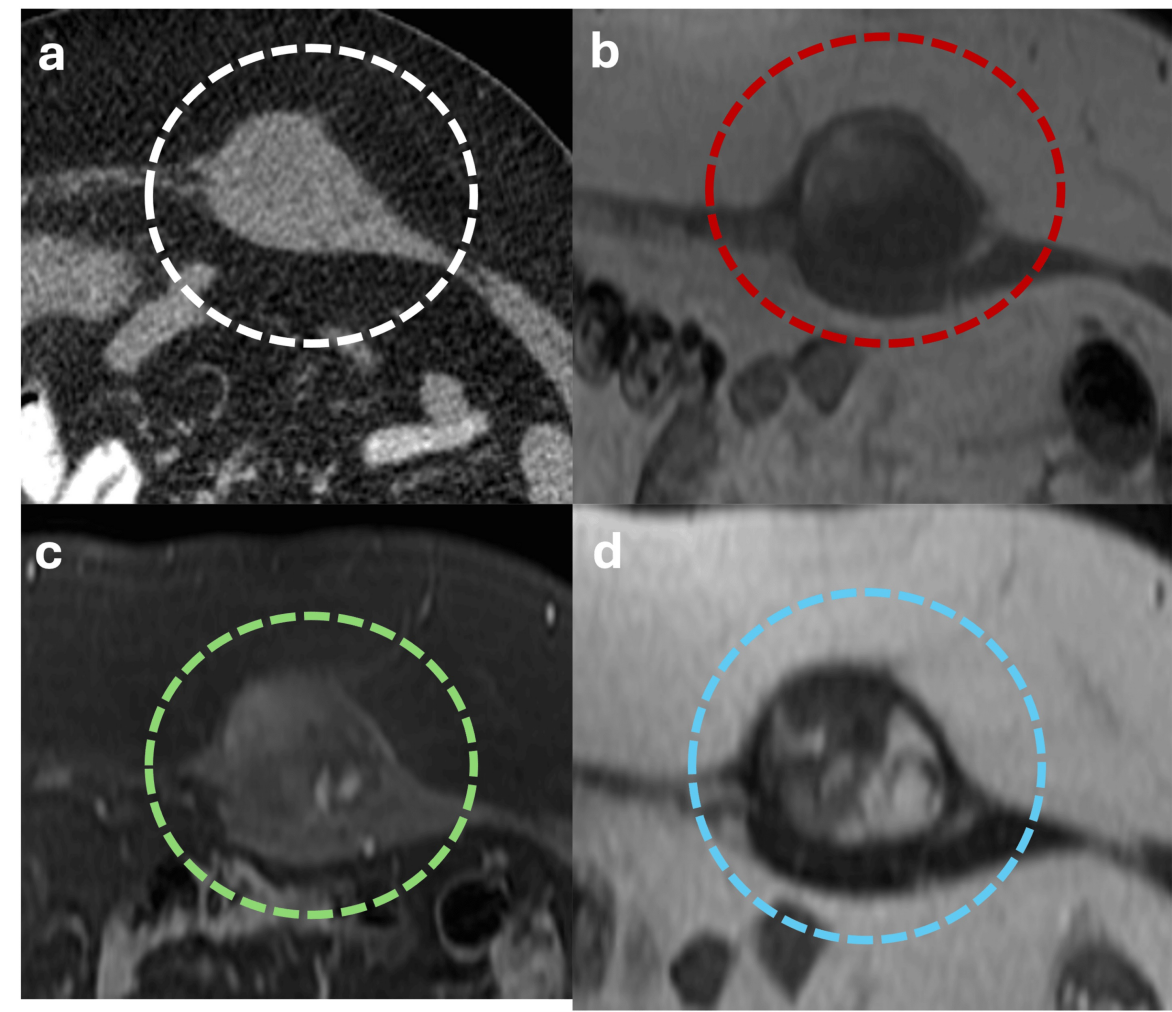
Axial contrast-enhanced abdominal CT and MRI showing a desmoid tumor (a) Axial CT reveals a soft-tissue density lesion with heterogeneous content and regular borders in the anterior abdominal wall (white dotted circle). (b, c) Axial MRI showing the same lesion depicting a T1 hypointense signal with poor contrast enhancement after contrast medium administration (red and green dotted circles, respectively); (d) the lesion exhibits a T2 hypointense signal with focal areas of high signal intensity due to cellular myxoid content (blue dotted circle), these findings collectively are consistent with desmoid tumor. The figure was created by the authors.

Desmoid tumor of the abdominal wall is a locally aggressive fibrous tumor with high recurrence rates, occurring more frequently in women than in men, with a ratio of 11:1. It can be associated with Gardner syndrome [20]. On CT scans, these tumors appear solid with variable contrast enhancement. MRI findings typically show T1 hypointensity, T2 hypointensity, and delayed enhancement on T1-weighted contrast-enhanced images. However, tumors exhibiting active growth may present focal areas of T2-weighted high signal-intensity due to high cellular-myxoid content (Figure 21) [20].

Abdominal‑wall endometriosis should remain on the differential diagnosis when a presumed desmoid tumor is encountered, yet several clinical‑imaging clues can help distinguish the two. Endometriotic implants usually contain punctate high‑signal foci on T1‑weighted MRI representing intralesional hemorrhage, arise in young women, often occur at a prior cesarean‑section scar, and may cause cyclic pain that peaks during menstruation.

Abdominal Wall Metastases

**Figure 22:**
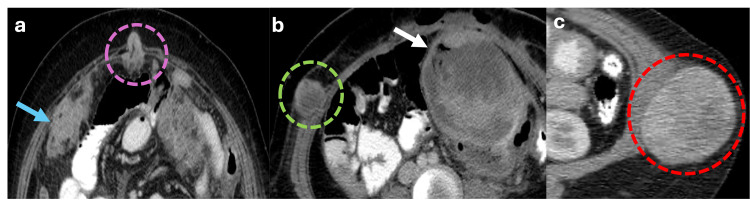
Axial abdominal CT scan with intravenous contrast showing a Sister Mary Joseph nodule, abdominal wall metastasis, and abdominal wall sarcoma (a) Axial CT scan shows a soft-tissue lesion with irregular borders around the umbilical region, also known as the sister Mary Joseph nodule (pink dotted circle), in a patient with advanced peritoneal carcinomatosis due to ovarian cancer (blue arrow). (b) Axial CT scan with intravenous contrast from another patient shows an irregular solid abdominal wall nodule with a necrotic core (green dotted circle) consistent with a metastatic deposit in a patient with a gastrointestinal stromal tumor (GIST) (white arrow). (c) Axial CT scan with intravenous contrast reveals a large oval-shaped mass with smooth borders and heterogeneous contrast enhancement located in the left lateral abdominal wall, partially invading the intramuscular structures, these findings in the context of a patient with no other neoplastic lesions is highly suggestive of a primary sarcoma (red dotted circle). The figure was created by the authors.

Metastases are the most common malignant tumors affecting the abdominal wall [20]. On CT scans, they appear as soft tissue masses with irregular borders that enhance with contrast medium (Figure 22a, 22b). Such masses are typically observed in advanced oncological patients. Hematogenous spread can occur predominantly from melanoma, lung carcinoma, and breast carcinoma. The direct extension from intra-abdominal tumors is another route of dissemination. Melanoma metastases depict high signal intensity in T1-weighted sequences due to melanin content and also have a higher tendency to bleed [20].

Abdominal Wall Sarcoma

Soft-tissue sarcomas are a diverse group of rare mesenchymal malignancies that can occur throughout the body. Various types of sarcomas target the abdominal wall, each displaying distinct demographic patterns [22]. Leiomyosarcoma is frequently observed in middle-aged men between 50 and 70 years, while dermatofibrosarcoma protuberans predominantly affects young brunette women. Rhabdomyosarcoma is primarily seen in children, epithelioid angiosarcoma tends to manifest in obese individuals, and synovial sarcoma is more common in adolescents and young adults. However, despite these demographic associations, diagnostic imaging cannot reliably differentiate between these different types of abdominal wall sarcomas (Figure 22c) [22].

Both abdominal‑wall metastasis and primary sarcomas often show similar imaging characteristics; distinguishing one from the other can be difficult. Sarcomas typically are larger (>5 cm), invade adjacent structures, and are detected in patients without known oncological disease.

Postsurgical changes in the abdominal wall

Abdominoplasty

**Figure 23:**
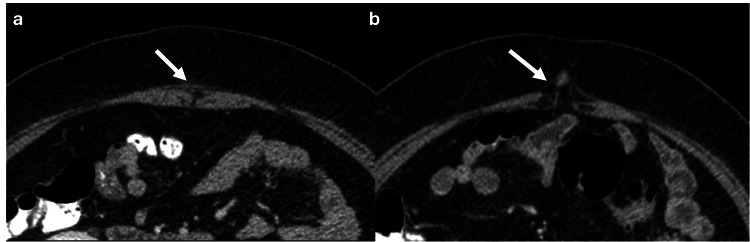
Axial abdominal CT scan with intravenous contrast showing post-abdominoplasty surgical changes (a) Axial CT scan shows the rectus abdominis muscles clumped along the midline (white arrow). (b) The umbilicus is remodeled, resembling a small tent (white arrow), these findings are consistent with post-abdominoplasty surgical changes. The figure was created by the authors.

Abdominoplasty is a cosmetic procedure designed to create a flatter and firmer abdomen [25]. The standard technique involves making a horizontal incision just above the pubic area, extending from hip to hip [25]. The umbilicus is separated from the surrounding skin, excess skin and fat are removed, and the rectus abdominis muscles are reapproximated with sutures to improve abdominal contour [25]. On CT imaging, the rectus abdominis muscles may appear contiguous along the midline, and the remodeled umbilicus can resemble a small tent (Figure 23) [25]. Common complications of abdominoplasty include seromas (10%), hematomas, skin flap necrosis, and, more rarely, pulmonary embolism [25].

Abdominal Wall Meshes

**Figure 24:**
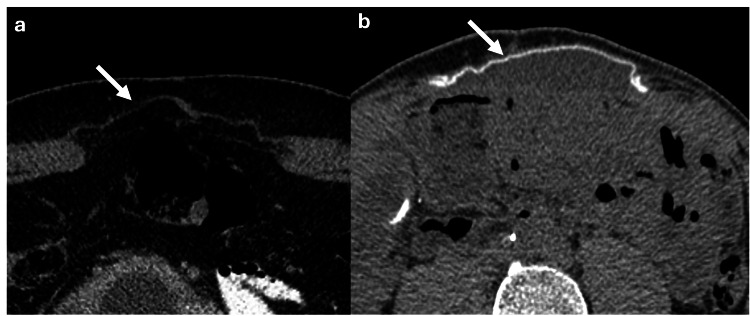
Axial abdominal CT scan without intravenous contrast. (a) Axial CT shows a polypropylene mesh in the anterior abdominal wall that is isodense to the surrounding muscle (white arrow). (b) Axial non contrast CT scan from a different patient with a PTFE mesh in the anterior abdominal wall that is hyperdense to the muscle, allowing better visualization on CT scans (white arrow). The figure was created by the authors.

Abdominal wall meshes are routinely used to repair most abdominal wall hernias [6]. Two primary types of meshes are commonly used: polypropylene and polytetrafluoroethylene (PTFE) meshes [6]. On CT imaging, polypropylene meshes are usually 0.44mm thick and appear isodense with the surrounding tissues, blending in with the normal anatomy [6]. By contrast, PTFE meshes are 1mm thick and are hyperdense, allowing for easier visualization on CT scans (Figure 24) (6]. The main complications associated with meshes are hernia recurrence (7%), seromas, hematomas, abscesses, and mesh shrinkage due to extensive surrounding fibrosis [6].

Buried Bumper Syndrome

**Figure 25:**
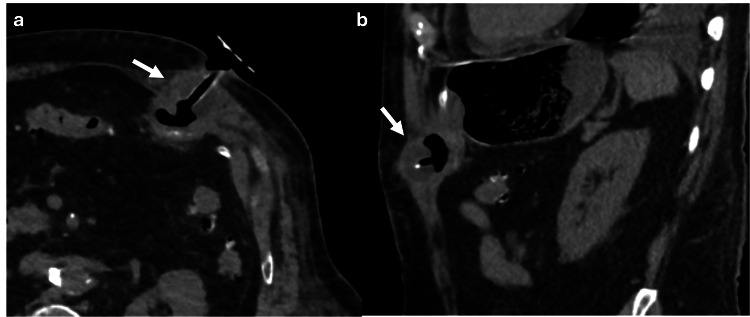
Axial non-enhanced abdominal CT scan showing a buried bumper syndrome (a, b) A 91-year-old male with a history of gastrostomy tube insertion presents with gastrointestinal bleeding and abdominal pain; notice the migration of the gastrostomy balloon and internal bumper into the rectus abdominis muscle (white arrows), leading to entrapment of the gastric mucosa, these findings collectively are suggestive of buried bumper syndrome. The figure was created by the authors.

Buried bumper syndrome occurs when the internal bumper of a gastrostomy tube migrates into the abdominal wall, leading to compression and entrapment of the gastric mucosa (Figure 25) [26]. This condition can result in gastric ischemia and necrosis [26]. Clinically, it presents with symptoms such as abdominal pain, leakage of gastric contents around the gastrostomy site, gastrointestinal bleeding, abscess formation, and, in severe cases, peritonitis [26]. Early recognition and management are essential to prevent serious complications.

## Conclusions

The abdominal wall is a complex structure susceptible to a variety of pathologies, including hernias, infections, and tumors. Radiologists must be familiar with the imaging features of common abdominal wall hernias, including inguinal, femoral, ventral, Spigelian, lumbar (Grynfelt and Petit), obturator, and rare types such as Amyand, Richter, and Garengeot hernias. In the past two decades, advances in surgical techniques have introduced new imaging challenges, making it essential for radiologists to recognize common and uncommon post-surgical changes in the abdominal wall. This pictorial review highlights key abdominal wall pathologies and their appearances on cross-sectional imaging, particularly CT and MRI, to provide radiologists with the knowledge needed for accurate diagnosis and effective patient management. Understanding anatomical landmarks, due to their high spatial resolution and soft-tissue contrast, recognizing imaging patterns, and the potential for complications significantly enhances diagnostic precision.
